# Treatment outcome in HIV+ patients receiving 3- or 4-drug regimens during PHI

**DOI:** 10.7448/IAS.17.4.19778

**Published:** 2014-11-02

**Authors:** Giulia Maria Bottani, Maria Letizia Oreni, Giancarlo Orofino, Pamela Tau, Silvia Di Nardo Stuppino, Elisa Colella, Sinibaldo Carosella, Marta Guastavigna, Valeria Ghisetti, Valeria Micheli, Massimo Galli, Stefano Rusconi

**Affiliations:** 1 Infectious Diseases Unit, DIBIC Luigi Sacco, Milan, Italy; 2 Infectious Diseases Unit A, Amedeo di Savoia Hospital, Turin, Italy; 3 Microbiology and Virology Laboratory, Amedeo di Savoia Hospital, Turin, Italy; 4 Clinical Microbiology, Virology and Bioemergency, Luigi Sacco Hospital, Milan, Italy

## Abstract

**Introduction:**

The optimal timing and modality of therapeutic intervention during early phases of HIV infection is still debated; in our prospective observational study we evaluated immunological and virological outcome in HIV+ patients treated during acute or recent HIV infection.

**Materials and Methods:**

A total of 25 naïve patients with acute (detectable HIV-RNA, immature Western Blot) or recent (documented infection within six months) HIV infection were recruited at the Infectious Diseases Units of the University of Milan and Turin from 2009 to 2014. Patients received treatment with two NRTIs+one NNRTI/bPI, with or without an induction phase with an additional fourth drug (raltegravir or maraviroc) until HIV-RNA undetectability maintained for six months. Blood samples for HIV-RNA, lymphocyte subsets and tropism assessment were obtained at the beginning of the treatment (BL). Patients underwent subsequent six-monthly follow up for clinical outcome, CD4 cell count and HIV-RNA up to 18 months.

**Results:**

Median increase in CD4 cells from 0 to 12 months was greater in patients treated during acute (n=18) versus recent (n=7) infection [284/µL, IQR (227–456) versus 176/µL, IQR (70–235); Mann-Whitney test, p=0.046]. This higher value was maintained through 18 months, although failing to reach statistical significance. Patients with acute or recent infection did not significantly differ in virological success (83.3% versus 85.7% at 12 months). We considered CD4 cells gains at six months (multivariate analysis, ANCOVA; Figure 1) and detected an inverse correlation with CD4 levels at BL (r=−0.517; p=0.008) and a direct correlation with the status of acute infection (r=0.234, p NS). This last correlation reached statistical significance at 12 months (r=0.418, p=0.035), whereas the inverse correlation with CD4 levels at BL was still present without a statistical significance (r=−0.350; p=0.072). Patients treated with three or four drugs did not show any significant difference in immunological nor virological response (Mann-Whitney and χ^2^ test). Modification or interruption of therapy for tolerability took place in 4 out of 25 patients, all while receiving four drugs; two patients underwent STI between 12 and 18 months following virological success.

**Conclusions:**

Treatment of primary infection appeared to be effective in preserving the pool of CD4 cells in acute more than recent infection. There was no evidence of a different outcome through the addition of a fourth drug to the standard treatment.

**Figure 1:**
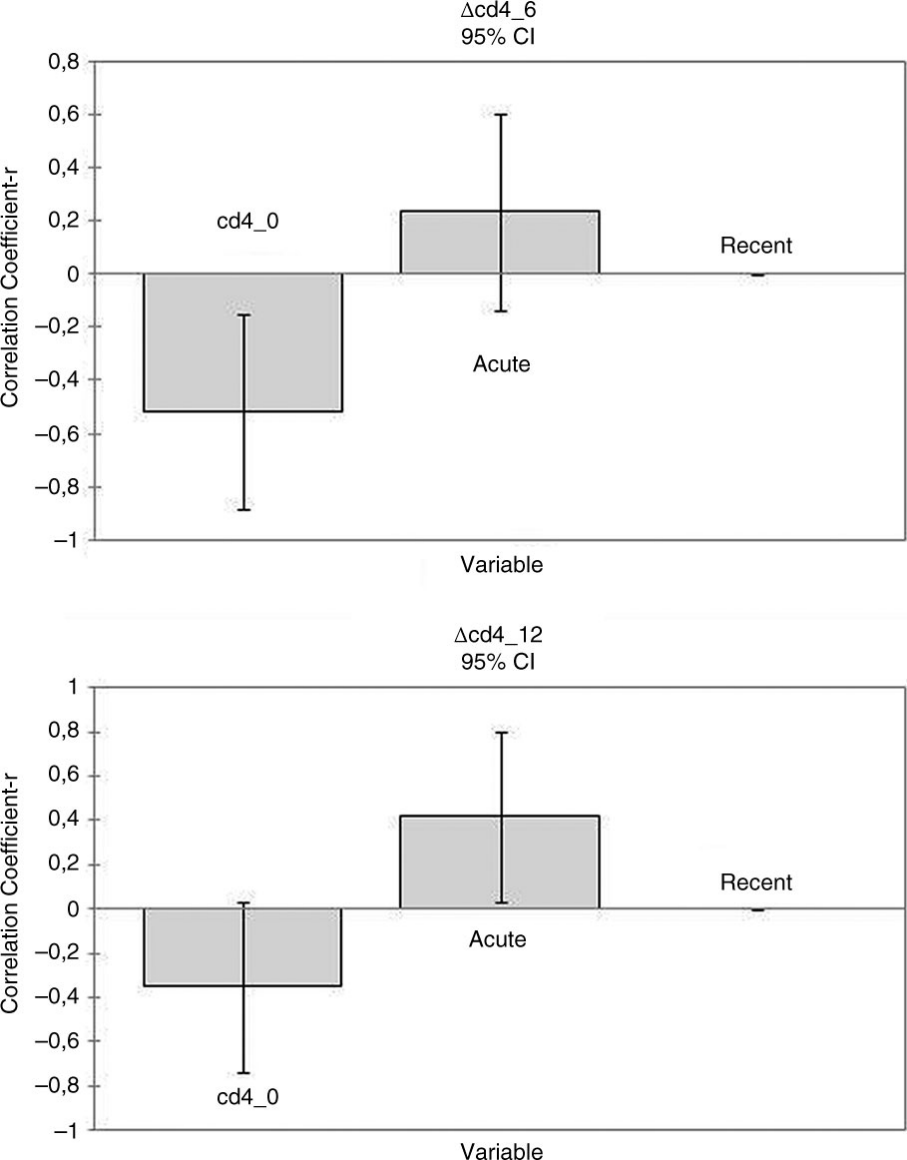
CD4/CL recovery among BL and months 6 and 12.

